# Research on the Adsorption Mechanism and Performance of Cotton Stalk-Based Biochar

**DOI:** 10.3390/molecules29245841

**Published:** 2024-12-11

**Authors:** Qiushuang Cui, Yong Huang, Xufei Ma, Sining Li, Ruyun Bai, Huan Li, Wen Liu, Hanyu Wei

**Affiliations:** 1 State Key Laboratory of Chemistry and Utilization of Carbon Based Energy Resources, College of Chemistry, Xinjiang University, Urumqi 830017, China; cuiqiushuang1212@163.com (Q.C.); bairu666666@163.com (R.B.); lh15809434726@163.com (H.L.); liuwen20000324@163.com (W.L.); weihycn@outlook.com (H.W.); 2 College of Civil Engineering and Architecture, Xinjiang University, Urumqi 830017, China; 3 Department of Chemistry and Applied Chemistry, Changji University, Changji 831110, China; xfma1225@163.com

**Keywords:** methylene blue, tetracycline, biochar, adsorption, KOH modification

## Abstract

In this research, we produced two types of biochar (BC) using cotton stalks as raw material and KOH as an activator, and compared their performance and adsorption mechanisms in the removal of tetracycline (TC) and methylene blue (MB) from wastewater. The results showed that the biochar generated using both procedures formed pores that connected to the interior of the biochar and had extensive microporous and mesoporous structures. The molten salt approach produces biochar with a higher specific surface area, larger pore size, and higher pore volume than the impregnation method, with a maximum specific surface area of 3095 m^2^/g. KBCM-900 (the BC produced using the molten salt method at 900 °C) had a better adsorption effect on TC, with a clearance rate of more than 95% in 180 min and a maximum adsorption amount of 912.212 mg/g. The adsorption rates of the two BCs for MB did not differ significantly at low concentrations, but as the concentration increased, KBCI-900 (the BC generated by the impregnation method at 900 °C) exhibited better adsorption, with a maximum adsorption of 723.726 mg/g. The pseudo-second-order kinetic model and the Langmuir isotherm model may accurately describe the TC and MB adsorption processes of KBCI-900 and KBCM-900. The KBCI/KBCM-900 adsorption process combines physical and chemical adsorption, with the primary mechanisms being pore filling, π–π interactions, hydrogen bonding, and electrostatic interactions. As a result, biochar generated using the molten salt method is suitable for the removal of large-molecule pollutants such as TC, whereas biochar prepared using the impregnation method is suitable for the removal of small-molecule dyes such as MB.

## 1. Introduction

As the world’s population continues to rise, the dyeing and farming industries have grown. This has resulted in a significant increase in the use of dyes and antibiotics, some of which are discharged untreated into the environment, posing an increasing threat to human health and aquatic ecosystems [1]. Tetracycline (TC) has been widely utilized in the treatment of bacterial infections in humans and animals due to its good antibacterial capabilities, minimal side effects, and inexpensive cost. However, research has shown that the majority of unmetabolized TC enter water bodies via excreta or direct discharges, constituting an important source of pollution and posing a substantial hazard to both the environment and public health [2,3]. Methylene blue (MB), a commonly used dye in the textile, leather, and paper industries, dissolves rapidly in water because it does not cling entirely to fibers [4]. As a result, MB is prone to discharge into bodies of water, providing a safety risk to aquatic organisms as well as human public health [5]. Furthermore, the discharge of MB limits sunlight transmission to water bodies and depletes the dissolved oxygen in water, putting aquatic creatures’ survival at risk [6]. To protect the environment and public health, it is critical to reduce and prevent the discharge of dye- and antibiotic-contaminated wastewater into natural water bodies [7,8].

The present approaches for treating this type of wastewater include advanced oxidation [9,10], photocatalysis [11], adsorption [12], membrane filtration [13], and the use of activated sludge [14]. Adsorption is regarded as a cost-effective technology for effectively removing harmful contaminants from water due to its ease of use, low cost, high adsorption capacity, low energy consumption, and environmental friendliness [15]. Furthermore, adsorbent biochar (BC) materials made from agricultural waste have gained popularity due to their environmentally friendly properties [16,17].

BC is a widely available, environmentally friendly, and efficient adsorbent [18,19] that is primarily generated from the pyrolysis of agricultural wastes [20], as well as commonly used biomasses such as cotton stalks [21], cornstalks, straw [22], walnut shells [23], and others, in an inert gas atmosphere. It has attracted a lot of interest because of its distinct physicochemical features, such as surface electronegativity and aromatization [24]. Biochar typically has a small specific surface area (SSA) and poor adsorption performance; however, appropriate treatment can improve its physicochemical qualities [25].

Frequently used biochar modifications include acid modification [26], alkali modification [27], salt modification [28], hydrothermal modification [29], and metal modification Park et al. [30] Qin et al. [31] examined the production of Napier grass biochar using 15% HNO_3_ and 40% KOH; the adsorption capacity of their biochar for methylene blue was enhanced to 101.13 mg/g when the pH was set to 1 and 15% HNO_3_ Was used. Min et al. [32] combined ZnCl_2_ impregnation and co-pyrolysis techniques to prepare biochar and found that ZnCl_2_ impregnation boosted the yield, elemental concentrations of O and S, aromaticity, and hydrophilicity of the resulting BC. In addition, ZnCl_2_ impregnation enhanced the percentage of aromatic C=C while decreasing the content of oxygenated functional groups (C=O and C-O). Liu et al. [33] modified walnut shell biochar (WSC) and wood flour biochar (WPC) with ZnCl_2_, KOH, H_2_SO_4_, and H_3_PO_4_. The strengths of the adsorption effects of the different treatment reagents on MB were in the order ZnCl_2_ > KOH > H_3_PO_4_ > H_2_SO_4_. The maximum adsorption capacities of the two biomass treatments were 850.9 mg/g for the WPC with ZnCl_2_ treatment and 701.3 mg/g for the WSC with KOH treatment. Md Azharul Islam et al. [34] activated rattan (*Lacosperma secundiflorum*) with NaOH to produce mesoporous activated carbon (HAC). Common adsorption variables resulted in a 96% removal effectiveness for an MB sample at an initial concentration of 25 mg/L, a solution pH of 7, and 30 °C for 8 h, with a maximum absorption of 359 mg/g for the obtained HAC, which had a high surface area of 1135 m^2^/g. Alkali-modified biochar has a higher SSA [35], richer pore structure, and better adsorption capability than other biochars seen in the literature. Furthermore, when compared to other modifications using metal ions or metal oxides, the alkali treatment process is more efficient and cannot result in secondary contamination [22].

At the moment, there are mainly two methods of alkali treatment: impregnation and molten salt. Shi et al. [23] found that KOH molten salt-treated walnut shell biochar pyrolyzed at 900 °C (KWS900) had a considerable increase in SSA compared to virgin walnut shells, equal to 1713 ± 37 m^2^/g, while the highest adsorption of TC by KWS900 was 607 ± 31 m^2^/g. Qu et al. [35] found that biochar generated from corn stover treated with KOH molten salt had an SSA of 2183 m^2^/g and many micropores, with an average pore size of 2.75 nm. Cheng et al. [36] reported that the KOH activation of grapefruit peel resulted in a high adsorption capacity of BC-KOH for tetracycline (476.19 mg/g), oxytetracycline (407.5 mg/g), and chlortetracycline (555.56 mg/g) at room temperature. Zhao et al. [37] employed KOH impregnation to activate reed-based biochar as an adsorbent, and their results showed that the internal structure of the resulting biochar was loose and porous, that the SSA increased by 194.08 times, reaching 965.31 m^2^/g, and that the biochar’s TC adsorption capacity increased by more than 20 times after impregnation and activation. Ding et al. [38] investigated the alkali impregnation modification of sludge activated carbon and discovered that KOH impregnation modification was the most effective way to increase the TC adsorption by sludge biochar. After three cycles, the adsorption capacity was reduced by 5.94%, although it remained 4.5 times higher than that of unaltered sludge BC. Thotsaporn Somsiripan et al. [39] synthesized biochar by pyrolyzing empty oil palm fruit bunches using KOH impregnation, the resulting material of which demonstrated the maximal adsorption of MB, malachite green (MG), and rhodamine B (RhB) at 1123.57, 1315.36, and 982.87 mg/g, respectively. KOH has long been employed for its effective intercalation capacity and great catalytic effect on precursor gasification [23,27,35], which can improve the efficiency and quality of pore formation. The impacts of the two activation methods on the characteristics of biochar have not been investigated. More research is needed to determine whether the molten salt approach and the impregnation method create biochar with similar or equivalent qualities when the same biomass precursors, biomass to alkali ratios, treatment periods, and temperatures are utilized [28]. As the main cotton production center in China, Xinjiang generates millions of tons of cotton stalks every year, in addition to the cotton harvest [40]. Cotton stalks have a carbon content of more than 40%, making them an ideal raw material for the production of carbonaceous compounds [41]. As a result, research into carbonaceous adsorbents based on cotton stalks can not only maximize the use of cotton stalks but also effectively address the issue of environmental water pollution.

Therefore, in the present research, porous biochar was generated utilizing the impregnation and molten salt methods, with cotton stalks as biomass and KOH as an activator. To investigate the adsorption of TC and MB by biochar generated with a KOH/cotton straw mass ratio of 1.0, as well as the influence of varied pH levels and adsorption periods of the resulting solution on TC and MB adsorption and the generated BCs’ physicochemical parameters, the kinetics and thermodynamics of TC and MB adsorption by biochar were analyzed, and the adsorption process and mechanism of biochar generated using various methods were examined.

## 2. Result and Discussions

### 2.1. Characterization of Biochar

**Figure 1:**
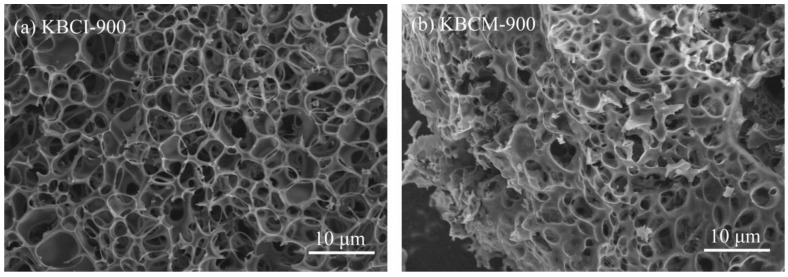
SEM of biochars: (**a**) KBCI-900; (**b**) KBCM-900.

Figure 1 shows SEM images of samples made using the impregnation and molten salt methods. As shown in Figure 1a, the surface of KBCI-900 exhibited rich and well-developed pores, while the crushed surface had internally connected pores. The formation of holes may be attributed to the reaction of the KOH with the biomass at high temperatures, which produces different levels of volatility. These volatiles comprised potassium metal vapors, CO, CO_2_, H_2_, and H_2_O, all of which promote the creation of micropores [42]. As can be seen in Figure 1b, KBCM-900 was discovered to be comparable to KBCI-900 in that it had a significant number of well-developed holes on its surface and within that were interconnected. This is because KOH remains highly corrosive, damaging the fibers of cotton stalks during the high-temperature reaction and acting as an etching agent, increasing the formation and development of holes [43].

**Figure 2:**
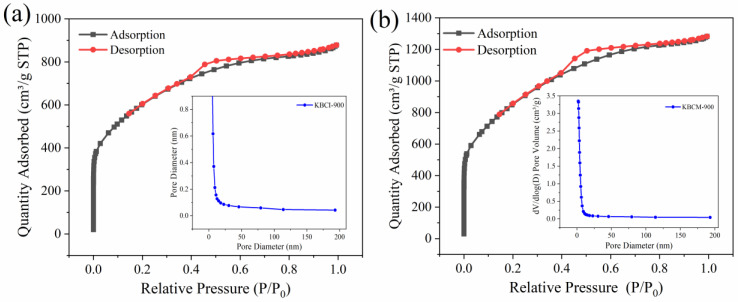
Nitrogen adsorption–desorption isotherms and pore size distribution of: (**a**) KBCI-900, (**b**) KBCM-900.

The SSA and pore structures of the samples generated using various procedures are displayed in Figure 2 and Table 1. In Figure 2a,b, the N_2_ adsorption–desorption isotherms of KBCI-900 and KBCM-900 were of type I and IV, respectively, with H4 hysteresis loops. The pore diameters of KBCI-900 and KBCM-900 were mainly distributed in the range of 2–50 nm, which indicates the formation of microporous and mesoporous structures in the carbon materials prepared by the two methods. As shown in Table 1, the SSA, pore diameter, and pore volume of KBCM-900 were larger than those of KBCI-900, and the SSA and pore volume of KBCM-900 were increased by 42.016% and 45.374%, respectively, relative to those of KBCI-900, indicating that the molten salt method improved the SSA and mesopore structure of the prepared biochar. After TC and MB adsorption with KBCI-900, the SSA fell from 2179 m^2^/g to 616 m^2^/g and 1140 m^2^/g, respectively, while the pore volume decreased from 1.36 cm^3^/g to 0.42 cm^3^/g and 0.76 cm^3^/g; after the adsorption of TC and MB with KBCM-900, the SSA decreased from 3095 m^2^/g to 725 m^2^/g and 1266 m^2^/g, respectively, and the pore volume decreased from 1.98 cm^3^/g to 0.45 cm^3^/g and 0.91 cm^3^/g, indicating that the pore filling of the two adsorbents played an important role in the adsorption process.

**Figure 3:**
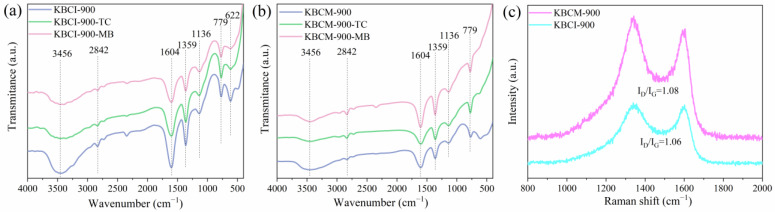
FTIR of KBCI/KBCM-900 before and after adsorption: (**a**) KBCI-900; (**b**) KBCM-900; (**c**) Raman plots of KBCI-900 and KBCM-900.

Figure 3 shows the FTIR results before and after the adsorption by the samples generated using the impregnation and molten salt procedures. Figure 3a,b shows that the absorption peaks at 3456 cm^−1^ are OH and NH stretched vibrations, whereas the nitrogen in the cotton stalks resulted in the production of nitrogen-containing functional groups [44]. The peak at 2842 cm^−1^ corresponds to the C-H bond stretching vibration caused by the cleavage of functional groups containing methyl, methylene, and oxygen, transforming the three-dimensional network of the benzene ring into the two-dimensional structure of the thick ring, which was then expanded and dehydrogenated to form the graphite microcrystalline structure [45]. The absorption peaks at 2351 cm^−1^ and 1359 cm^−1^ indicate C≡N and -NO_2_ bond stretching vibrations, respectively. The peak at 1604 cm^−1^ this corresponds to an aromatic C=C asymmetric stretching vibrational absorption, indicating a highly persistent aromatic structure due to KOH activation [46,47]. The absorption peak at 1136 cm^−1^ is the characteristic peak of ethers and esters (C-O) [48]. The band at 1000–650 cm^−1^ was identified as γ-CH in the aromatic ring, which can give π electrons to form π–π bonds with TC^+^ and MB^+^, promoting MB and TC adsorption [45]. The functional group’s composition was not significantly altered with TC and MB adsorption, although the peak intensity did. This could be due to the fact that the principal functional groups of KBCI-900 and KBCM-900 were similar to those of TC and MB, resulting in modifications to the content of certain functional groups following adsorption.

The Raman spectroscopy (Figure 3c) revealed the structural characteristics of biogenic carbon, with KBCI-900 and KBCM-900 exhibiting spectral bands at 1350 cm^−1^ and 1590 cm^−1^, corresponding to defective sites or disordered sp2 hybridized carbon (D band) and a crystalline graphitic structure in porous carbon (G band), respectively. As shown in the figure, the ID/IG value of KBI-900 (1.06) was slightly lower than that of KBCM-900 (1.08), indicating that KBCM-900 was defective and had a low degree of graphitization.

**Figure 4:**
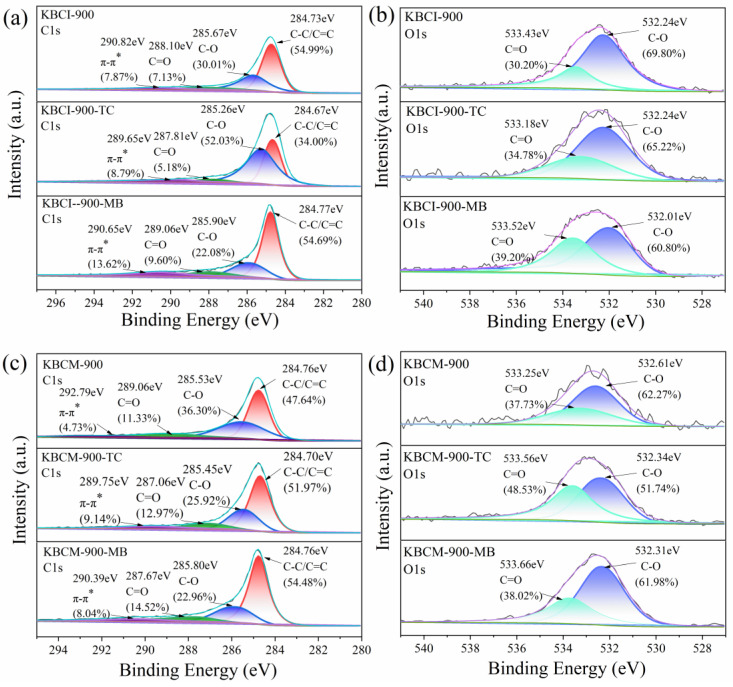
XPS images of BC: (**a**,**b**) KBCI-900 before and after adsorption; (**c**,**d**) KBCM-900 before and after adsorption.

Figure 4 depicts the XPS images obtained before and after the TC and MB adsorption by KBCI-900 and KBCM-900. As shown in Figure 4a, as indicated by the C 1s spectra, KBCI-900 exhibited four peaks at 284.73 eV, 285.67 eV, 288.10 eV, and 290.82 eV, which represent C-C/C=C, C-O, C=O, and π–π* bonds, respectively. After the adsorption of TC and MB by KBCI-900, the area of π–π* bond peaks rose from 7.87% to 8.79% and 13.62%, respectively, and both π–π* bonds shifted to lower binding energies, most likely due to π–π stacking. As shown in Figure 4b, the O 1s spectra showed two peaks at 532.24 eV and 533.43 eV, corresponding to C-O and C=O bonds, respectively, and the content of both C-O and C=O bonds influenced the following adsorption, indicating that hydrogen bonding is important in the TC and MB adsorption processes. As shown in Figure 4c, the C 1s spectra of KBCM-900 were comparable to those of KBCI-900, with an increase in the π–π* bond peak area due to π–π stacking after TC and MB adsorption. The fitted O 1s spectra of KBCM-900, shown in Figure 4d, showed that the C-O bond peak area of KBCM-900 dropped while the C=O bond peak area rose, indicating that hydrogen bonding is involved in the KBCM-900 adsorption process.

### 2.2. Adsorption Experiments

#### 2.2.1. Adsorption Properties of Biochar on TC and MB

**Figure 5:**
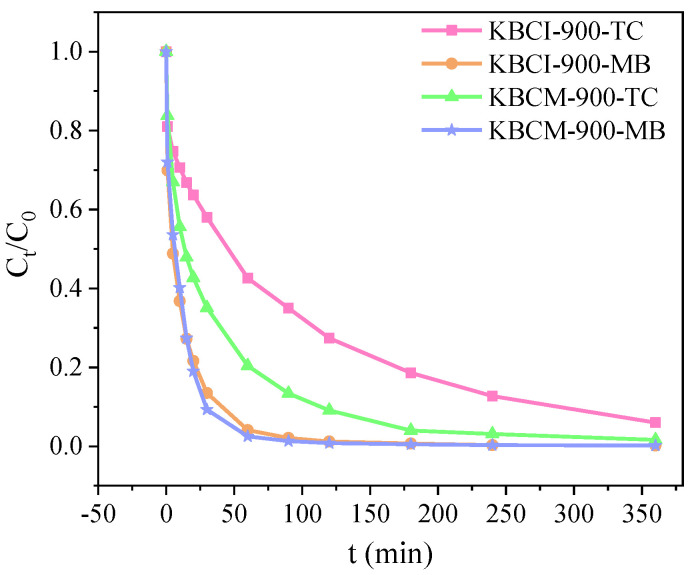
Adsorption performance of KBCI-900 and KBCM-900 on TC and MB.

Figure 5 illustrates the adsorption performances of KBCI-900 and KBCM-900 for TC and MB. As illustrated in Figure 5, the adsorption rates of KBCI-900 and KBCM-900 for TC, for 180 min under the identical circumstances, were 81% and 96%, respectively. They also absorbed 93% and 95% of the MB, respectively. The adsorption efficiency of KBCM-900 for TC was higher than that of KBCI-900 because the diameter of the TC was about 1.27 nm and the mesopore played a dominant role in the adsorption process of TC, whereas the mesopore pore size, SSA, and pore volume of KBCM-900 were higher than those of KBCI-900, and thus the adsorption of TC was better with KBCM-900. The faster adsorption rate of the adsorbents for MB compared to TC could be attributed to MB’s smaller diameter, which allows it to pass more easily through micropores. And while there is a significant variation in pore size and pore volume between the two types of biochar SSA, the difference in their adsorption performance is minimal; therefore, the adsorption performance is unrelated to pore size and SSA.

#### 2.2.2. The Influence of pH on Adsorption Effect

**Figure 6:**
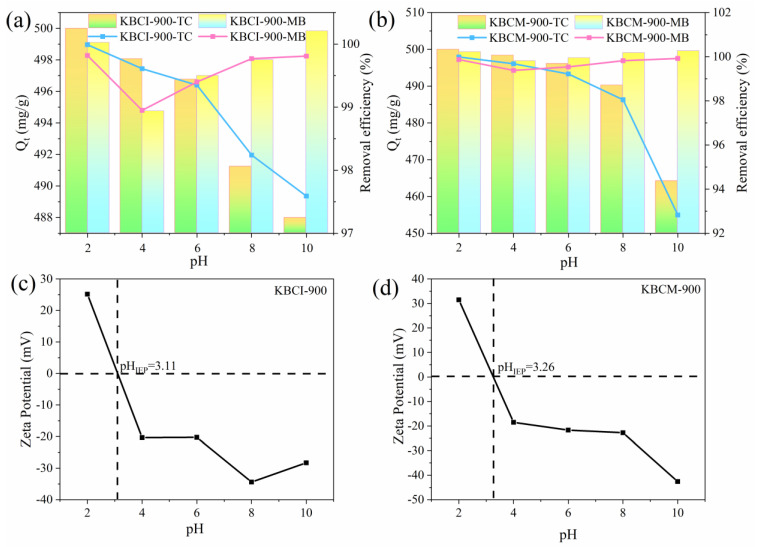
(**a**,**b**) Adsorption properties at different pH values; (**c**,**d**) zeta potentials.

Figure 6 depicts the adsorption performance graphs and zeta potentials of KBCI-900 and KBCM-900 for TC and MB at various pH levels. Figure 6a,b shows that, as the pH increased, the trend of MB and TC adsorption by the biochar changed, first decreasing and then increasing, which could be attributed to the presence of H^+^ while MB was positively charged, and the surface of the adsorbent also being positively charged, resulting in repulsion and thus decreasing the amount of adsorption. According to Figure 6c,d, the pH_IEP_ values of KBCI-900 and KBCM-900 were 3.11 and 3.26, respectively. When the pH was higher than the adsorbent’s pH_IEP_, the adsorbent’s surface became negatively charged, and the electrostatic attraction between the adsorbent and the MB eventually took over, increasing the adsorption rate. When the pH is lower than the adsorbent’s pH_IEP_, TC exists in the solution as TC^+^, the adsorbent’s surface is protonated and thus positively charged, and the adsorbent is electrostatically repelled from the TC [49], causing the adsorption rate to decrease. When the pH is higher than the pH_IEP_ of the adsorbent, the polar functional groups of the adsorbent segregate and become negatively charged, the TC is converted to TC(TC^−^) [50,51], the electrostatic repulsion continues to increase, and the adsorption continues to decrease. At this point, there was still a significant amount of adsorption, showing that the hydrogen bonding between TC and KBCM-900 through π–π interaction remained dominant.

#### 2.2.3. Adsorption Kinetic Models

**Figure 7:**
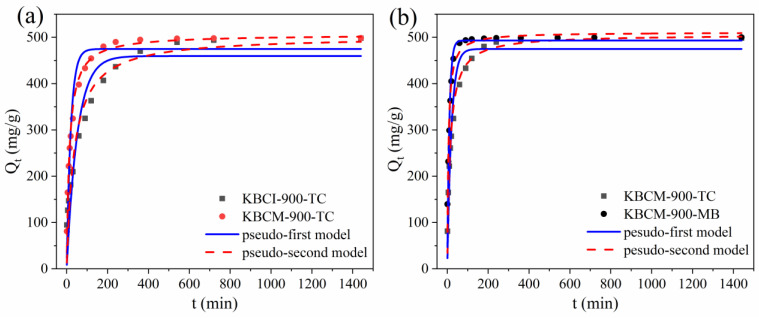
Kinetic modeling of TC and MB adsorption of biochar: (**a**) KBCI-900; (**b**) KBCM-900.

To further investigate the adsorption mechanism, the data were fitted using the PFO, PSO, and Webber–Morris models; the results are shown in Figure 7, and the kinetic fitting parameters are shown in Table 2. Figure 7a,b shows that the adsorption quantities for TC were identical, while KBCM-900 reached the adsorption equilibrium before KBCI-900. Both KBCI-900 and KBCM-900 demonstrated excellent MB adsorption capabilities, with similar adsorption equilibriums and adsorption quantities. As shown in Table 2, the pseudo-second-order model had greater correlation coefficients with the KBCI-900 and KBCM-900 than the pseudo-first-order model, indicating that the pseudo-second-order model was more suited for characterizing the adsorption kinetics of the studied materials for TC and MB. The pseudo-second-order model’s Q_e_ values (479.925 mg/g, 497.883 mg/g, 498.283 mg/g, 498.521 mg/g) were closer to the experimental values (498.073 mg/g, 499.971 mg/g, 498.399 mg/g, 499.875 mg/g). This indicates that the adsorption of TC and MB by BC includes chemical adsorption processes that involve electron sharing or exchange, such as H-bonding, complexation, and ion exchange [52,53].

**Figure 8:**
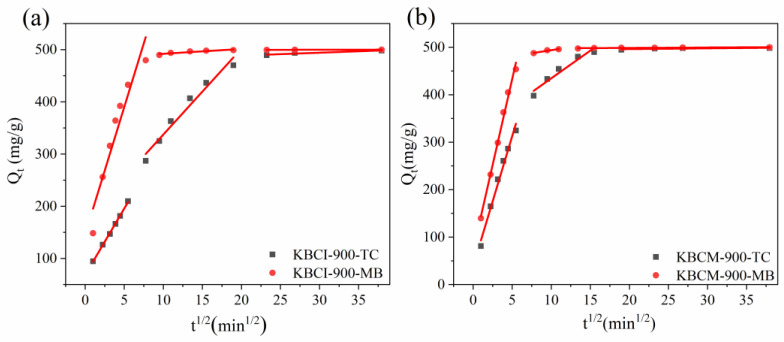
BC to TC and MB internal diffusion model plots: (**a**) KBCI-900; (**b**) KBCM-900.

In order to better investigate the kinematic characteristics of the adsorption process, the experimental data were analyzed using the intra-particle diffusion model, the findings of which are displayed in Figure 8 and Table 3. The fitting findings revealed that both adsorbents, for TC and MB, had a three-stage adsorption procedure. The first stage was surface diffusion, in which TC(MB) molecules diffused from the solution to the adsorbent surface due to the large number of adsorption sites and the rapid rate of adsorption; the second stage involved internal mesopore diffusion, which slowed down the adsorption by occupying surface adsorption sites. The rate constants and C values of both adsorbents followed the order of K_int1_ > K_int2_ > K_int3_ and C_1_ < C_2_ < C_3_, indicating that internal diffusion within the particles was the primary rate-controlling step; the third stage was intra-micropore diffusion, for which the low K_int3_ value indicated that the adsorption had gradually reached equilibrium due to the slow rate of diffusion in the pores caused by the low concentration of TC(MB) in the solution [54].

#### 2.2.4. Adsorption Isotherm Models

**Figure 9:**
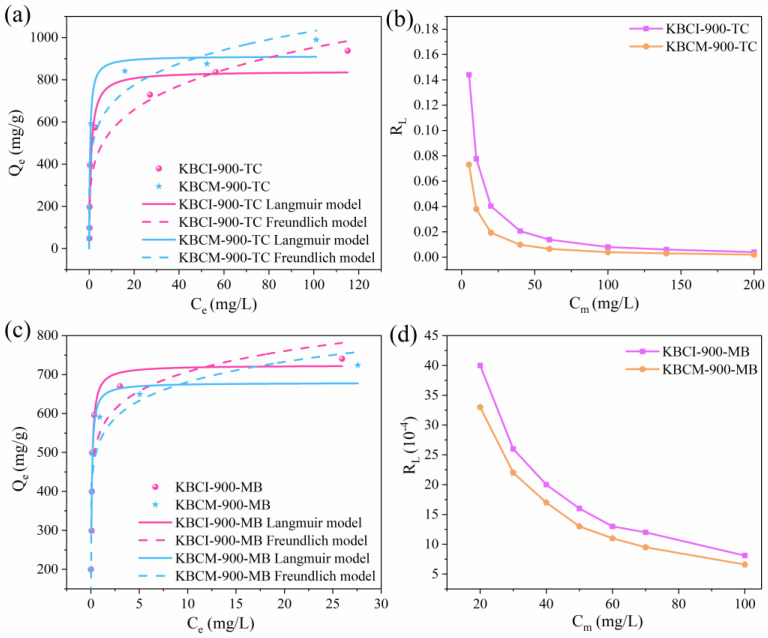
(**a**,**c**) Adsorption isotherms of TC and MB on KBCI-900 and KBCM-900; (**b**,**d**) plots of separation factor (RL) versus initial TC concentration.

The Langmuir, Freundlich, and D–R isotherm models were used to fit the experimental data and predict the maximum adsorption capacities for TC and MB, as well as the adsorption features of the two biochars. The adsorption of KBCI-900 and KBCM-900 increased rapidly and approached equilibrium with increasing initial TC and MB concentrations (Figure 9a,b). This tendency arises because higher starting concentrations give a greater pushing force to overcome the impedance to mass transfer between the solvent and solute surfaces [55]. The Langmuir model indicates that the adsorption behavior on the adsorbent is that of monolayers, whereas the Freundlich model predicts that the desorption behavior happens on heterogeneous surfaces with unequal binding sites. For both BCs, the Langmuir model fit better than the Freundlich model, indicating that KBCI-900 and KBCM-900 were monolayer adsorbants for TC and MB. The maximal TC absorptions by KBCI-900 and KBCM-900 were calculated to be 840.500 mg/g and 912.212 mg/g for TC and 723.726 mg/g and 678.904 mg/g for MB, respectively, using the Langmuir isotherm model (Table 4). During the adsorption of TC and MB by KBCI-900 and KBCM-900, the RL values ranged between 0 and 1 (Figure 9c,d), indicating that the surfaces of the two adsorbents favored TC and MB absorption [56].

In using Freundlich model, we employed n_F_ to determine the kind of adsorption: physisorption (n_F_ > 1), chemisorption (n_F_ < 1), or linear adsorption (n_F_ = 1). The n_F_s of the two BCs for TC and MB were 4.346, 9.546, 5.635, and 9.458, indicating a physisorption process. Furthermore, the ratio 1/n_F_ is directly related to surface heterogeneity, with the value of the surface heterogeneity being closer to zero the more inhomogeneous the surface of the material is [57]. The 1/n_F_ values of the Freundlich equation for the adsorption of TC and MB by KBI-900 were 0.230 and 0.105, respectively, and those of KBCM-900 for TC and MB were 0.177 and 0.106, respectively, with the value of the 1/n_F_ of KBCM-900 being closer to 0, indicating that the surface of KBCM-900 was more inhomogeneous. Furthermore, the adsorption energy (E) predicted with the D–R model for all BCs were less than 8 kJ/mol (Table 4, Figure S1), indicating that both the TC and MB adsorbents underwent physical adsorption.

#### 2.2.5. Regenerative Adsorption

The regeneration performance is a key index for evaluating biochar adsorption performance; consequently, the regeneration performance of both KBCI-900 and KBCM-900 was investigated, with the results provided in Figure S2. After the first, second, and third cycles, the adsorption efficiencies of KBCI-900 were determined to be 99.35%, 91%, and 50.64% for TC and 99.82%, 88.07%, and 69.93% for MB. KBCM-900 exhibited adsorption efficiencies of 99.29%, 96.35%, and 76.90% for TC and 99.84%, 77.00%, and 48.92% for MB, respectively. The desorption of MB by ethanol decreases the hydrophilicity of the adsorbent, which is a hydrophobic substance. Thus, the adsorption capacity of the adsorbent for MB decreases [8]. NaOH desorption causes the TC to release more charge and modifies the electrostatic contact between the TC and the adsorbent surface, decreasing the adsorption rate. After four cycles, KBCI-900’s adsorption performance on MB was 44.70% that of the first cycle. In contrast, the adsorption efficiency of KBCM-900 for TC after four cycles was 49.69% that of the first. Finally, both KBCI-900 and KBCM-900 performed well as reusable adsorbents.

## 3. Materials and Methods

### 3.1. Materials and Medicines

Cotton stalks were purchased from Xinjiang Kashi Farm (Kashi, China), while TC and MB (purity ≥ 98%) came from General-Reagent Company (Shanghai, China). The major elements of cotton stalks are shown in Table S1. Tianjin Zhiyuan Chemical Reagent Co. (Tianjin, China) provided anhydrous ethanol with a purity of 99.7% or higher. Other reagents, such as KOH, HCl, and NaOH, were purchased from General-Reagent Company with purity of AR. Deionized water was produced in the laboratory. Jinhongshan Company (Urumqi, China) provided N_2_ and common nitrogen with purity of ≥99.99%.

### 3.2. Biochar Preparation

Cotton stalks were soaked in deionized water for 16 h, dried at 105 °C, crushed in a pulverizer, sieved through an 80-mesh sieve, and left aside for modification using two methods. (1) Molten salt method: a mass ratio of 1:1 KOH and cotton stalks powder was placed in the mortar mixing and grinding, placed in the corundum ceramic boat, and then transferred to the tube furnace (Anhui Kemi, Hefei, China, TFH-1200-50-②-200) in a N_2_ atmosphere with a 5 °C/min rate of warming up to a specific temperature (900 °C). Finally, it was subjected to pyrolysis for 2 h to obtain KBCM-900. (2) Impregnation method: prepare a 10% KOH solution, weigh 1 g dry cotton stalks and immerse them in the solution, then dry them at 105 °C for 12 h, place them in a corundum porcelain boat, and transfer them to a tube furnace; the pyrolysis conditions were the same as those of the molten-salt method, and this sample was recorded as KBCI-900. Samples from various procedures were washed with distilled water, 0.1 mol/L hydrochloric acid, and 80 °C hot water until neutral, then dried at 105 °C for 12 h.

### 3.3. Characterization of Biochars

The synthesized BCs underwent SEM, FTIR, Raman, BET, and XPS characterization studies. SEM measurements were conducted using a field emission scanning electron microscope (Hitachi S4800, Hitachi, Tokyo, Japan), and gold was sprayed twice before to the test to evaluate microscopic morphology at a magnification of 2000 times. Fourier transform infrared (FTIR) spectroscopy (Great 10, CK Ruijie Technology Co., Tianjin, China) was used to assess the functional groups of BC using the potassium bromide press method over a range from 4000 cm^−1^ to 400 cm^−1^ with 32 scans. The valence states of biochar elements were determined using X-ray photoelectron spectroscopy (XPS; Thermo Fisher, Waltham, MA, USA). Biochar N_2_ adsorption–desorption isotherms, as well as specific surface area and pore size distribution, were determined using a gas adsorption meter (BET, Mack, South Allentown, PA, USA). The degree of carbon structural flaws and graphitization in biochar was studied using a confocal micro-Raman spectrometer (HORIBA, Longjumeau, France) with a test laser wavelength of 532 nm and a test range from 800 cm^−1^ to 2000 cm^−1^.

### 3.4. Biochar Adsorption Experiments on TC and MB

#### 3.4.1. Adsorption Performance Experiment

Five milligrams of BC was weighed and added to 50 mL of 50 mg/L TC(MB) solution, which was shaken (0, 1, 5, 10, 15, 20, 30, 60, 90, 120, 180, 240, 360 min) at a constant temperature under light-avoiding conditions. The supernatant was filtered, and the concentration of residual TC(MB) in the solution was measured using a UV-visible spectrophotometer (Essence 752, Shanghai Essence Technology Instrument Co., Ltd., Shanghai, China) at 356 nm and 664 nm. The adsorption and removal rates were determined using Equations (1) and (2), respectively:

(1) $$q_{t}=\frac{(C_{0}-C_{t})\times V}{m}$$

(2) $$Removal\;efficiency=\frac{C_{0}-C_{t}}{C_{0}}$$

where q_t_ and C_t_ represent the adsorbed amount and TC (MB) concentration at the prescribed time (t); C_0_, V, and m represent the initial concentration, volume, and adsorbance of TC (MB) in the reaction system, respectively.

#### 3.4.2. Effect of the Initial pH

A total of 5 mg of KBCI/KBCM-900 was weighed and added to 50 mg/L TC (MB) solution at various pH levels (pH = 2, 4, 6, 8, 10), and the experiment was carried out using the experimental procedure detailed in Section 3.4.1.

#### 3.4.3. Adsorption Kinetics Experiment

A total of 10 mg of BC was added to 100 mL of 50 mg/L TC (MB) solution, and the pH was adjusted to the appropriate value based on the preceding results. The residue concentration in the supernatant was then evaluated by shaking the conical flask at 180 rpm in a thermostatic shaker, protected from light, and obtaining samples at various periods (1, 2.5, 5, 10, 15, 20, 30, 60, 90, 120, 180, 240, 360, 540, 720, and 1440 min). The experimental findings were also fitted using the quasi-primary kinetic model, quasi-secondary kinetic model, and Webber–Morris model to explore the kinetic processes of adsorbents on MB and TC. The fitted equations are shown in Equations (S1)–(S3).

#### 3.4.4. Adsorption Isotherm Experiment

Combine 5 mg of KBCI/KBCM-900 with 50 mL of (5, 10, 20, 40, 60, 100, 140, 200 mg/L) TC solution (20, 30, 40, 50, 60, 70, 100 mg/L MB solution). The mixtures were agitated at 180 rpm for 24 h at 25 °C in the dark, and the residual concentration in the supernatant was measured. The data were fitted using three isothermal adsorption models: Langmuir, Freundlich, and Dubinin–Radushkevich (D–R). The fitted equations appear in Equations (S4)–(S9):

#### 3.4.5. Regenerative Adsorption Experiment

Recycle tests were carried out to investigate the regenerative properties of KBCI/KBCM-900. First, 10 mg KBCI/KBCM-900 was added to 100 mL of 50 mg/L TC (MB) solution for 24 h. The adsorbance was then measured, the adsorbent was recovered, and the MB was desorbed with anhydrous ethanol (0.2 mol/L NaOH solution for TC) for 24 h. The adsorbent was then removed from the sample. Finally, the samples were filtered and dried, and the experiment was performed four times.

## 4. Conclusions

In this paper, we investigated the adsorption performance, processes, and mechanisms of biochar synthesized using various methods for TC and MB adsorption at various pH values, concentrations, and times. Both methods produced biochar that had numerous interior pores, but the biochar generated from molten salt had a greater specific surface area, pore diameter, and pore volume. The two biochars had equivalent adsorption capacities for MB at low concentrations, with adsorption efficiencies exceeding 93%; the maximum adsorption of TC by the biochar produced by the molten salt method (912.212 mg/g) was higher than that of the biochar produced by the impregnation method (840.500 mg/g). The Langmuir model fitting showed that both biochars adsorbed both TC and MB monolayers. Adsorption involves chemisorption and physisorption, with the primary processes being π–π stacking, pore filling, hydrogen bonding, and electrostatic contact. Both biochars have a good regeneration cycle ability; after four cycles, the adsorption of TC and MB by the biochar generated by the impregnation method was 147.890 mg/g and 223.100 mg/g, respectively; the adsorption of TC and MB by the biochar generated by the molten salt method was 246.688 mg/g and 187.744 mg/g. Molten salt-modified biochar is more successful at removing TC from water, but impregnation-modified biochar is more effective at removing MB; thus, in practice, molten salt-modified biochar and impregnation-modified biochar have distinct effects and applications. This work focuses on the effects of the preparation methods of biochar on the adsorption of various contaminants, which is critical for the use of modified biochar and comprehending the challenges it faces.

## Figures and Tables

**Table 1:** KBCI/KBCM-900 sample SSA, microporous specific surface area, total pore volume/pore volume, microporous volume/pore volume, average pore diameter, and average pore diameter of mesopores.

Scheme 2.	SSA (m^2^/g)	Microporous Area (m^2^/g)	Total Pore Volume (cm^3^/g)	Microporous Volume (cm^3^/g)	Average Pore Diameter (nm)	Mesoporous Average Pore Size (nm)
KBCI-900	2179	1875	1.36	1.02	2.49	2.05
KBCM-900	3095	2611	1.98	1.48	2.56	3.09
KBCI-900-TC	616	525	0.42	0.30	2.69	2.27
KBCI-900-MB	1140	979	0.76	0.56	2.68	2.21
KBCM-900-TC	725	477	0.45	0.23	2.49	2.06
KBCM-900-MB	1266	1111	0.91	0.73	2.88	2.33

**Table 2:** Pseudo-first-order kinetic and pseudo-second-order kinetic model fitting parameters for the adsorption kinetics of TC and MB by biochar.

Sample	Q_e,exp_ (mg/g)	Pseudo-First-Order Model			Pseudo-Second-Order Model		
		k_1_ (min^−1^)	Q_e,cal_ (mg/g)	R^2^	K_2_ (min^−1^)	Q_e,cal_ (mg/g)	R^2^
KBCI-900-TC	498.073	0.019	459.507	0.889	7.555 × 10^−5^	479.925	0.947
KBCI-900-MB	499.971	0.108	487.995	0.899	4.049 × 10^−4^	497.883	0.968
KBCM-900-TC	498.399	0.050	474.749	0.937	1.446 × 10^−4^	498.283	0.984
KBCM-900-MB	499.875	0.100	493.094	0.938	3.616 × 10^−4^	498.521	0.967

**Table 3:** BC to TC and MB internal diffusion simulation ensemble parameters.

Sample	K_int1_ (mg/(g·min^0.5^))	C_1_	R^2^	K_int2_ (mg/(g·min^0.5^))	C_2_	R^2^	K_int3_ (mg/(g·min^0.5^))	C_3_	R^2^
KBCI-900-TC	25.453	68.439	0.999	15.457	172.751	0.968	0.547	477.677	0.908
KBCI-900-MB	48.708	146.622	0.916	0.921	482.867	0.844	0.028	498.939	0.875
KBCD-900-TC	54.895	38.263	0.985	11.687	317.665	0.943	0.158	492.954	0.622
KBCD-900-MB	72.268	71.774	0.993	2.566	468.216	0.957	0.084	496.939	0.789

**Table 4:** Adsorption isotherm parameters of TC and MB on KBCI-900 and KBCM-900.

Sample	Langmuir			Freundlich			Dubinin-Radushkevich	
	Qm (mg/g)	K_L_ (L/mg)	R^2^	K_F_ (mg/g)	n_F_	R^2^	E (KJ/mol)	R^2^
KBCI-900-TC	840.500	1.188	0.933	330.115	4.346	0.888	2.214	0.933
KBCI-900-MB	723.726	12.336	0.937	555.495	9.546	0.685	5.27	0.913
KBCM-900-TC	912.212	2.535	0.984	455.236	5.635	0.925	4.20	0.925
KBCM-900-MB	678.904	15.038	0.952	533.776	9.458	0.732	5.85	0.959

## Data Availability

The data presented in this study are available on request from the corresponding authors.

## Supplementary Materials

The following supporting information can be downloaded at: https://www.mdpi.com/article/10.3390/molecules29245841/s1. Table S1: Content of major elements in cotton stalks; Figure S1: D-R modeling plots of (a,b) KBCI-900 and (c,d) KBCM-900 for TC and MB; Figure S2: The 4 times of adsorption-desorption experiments by KBCI-900/KBCM-900.
