# Supplementary material for: Research on the Adsorption Mechanism and Performance of Cotton Stalk-Based Biochar
Source: Molecules. 2024 Dec 11;29(24):5841. doi: 10.3390/molecules29245841 (PMC11678485; doi:10.3390/molecules29245841)
Supplement: Supplementary file 1 [file molecules-29-05841-s001.zip › molecules-3340804-supplementary.pdf]

Research on the Adsorption Mechanism and Performance of Cotton Stalk-Based Biochar

Qiushuang Cui [1], Yong Huang [1,2,*], Xufei Ma [3], Sining Li [1,*], Ruyun Bai [1], Huan Li [1], Wen Liu [1] and Hanyu Wei [1]

1 State Key Laboratory of Chemistry and Utilization of Carbon Based Energy Resources, College of Chemistry, Xinjiang University, Urumqi 830017, China; cuiqiushuang1212@163.com (Q.C.); bairu666666@163.com (R.B.); lh15809434726@163.com (H.L.); liuwen20000324@163.com (W.L.); weihycn@outlook.com (H.W.)
2 College of Civil Engineering and Architecture, Xinjiang University, Urumqi 830017, China
3 Department of Chemistry and Applied Chemistry, Changji University, Changji 831110, China; xfma1225@163.com
* Correspondence: pengyou0991@163.com (Y.H.); lsn970525@163.com (S.L.)

1. Adsorption kinetics:

1.1 Pseudo-first-order:

$$\ln(Q_e - Q_t) = \ln Q_e - K_1 t \quad \text{(S1)}$$

Where K_1 (min^{-1}) was the pseudo-first order adsorption rate constant.

1.2 Pseudo-second order:

$$t/Q_t = 1/K_2 {Q_e}^2 + t/Q_e \quad \text{(S2)}$$

Where K_2 (g/(mg·min)) was the pseudo-second order adsorption rate constant.

1.3 Webber-Morris:

$$Q_t = K_i t^{1/2} + C_i \quad \text{(S3)}$$

Where K_i (mg/(g·$min^{0.5}$)) was the adsorption rate constants of intra-particle diffusion model and C_i was the constant for film thickness of the intra-particle diffusion model.

2. Adsorption isotherms:

2.1 Langmuir modal:

$$Qe = \frac{q_{max} K_L C_e}{1 + K_L C_e} \quad \text{(S4)}$$

Where K_L (L/mg) was the Langmuir constant, q_{max} (mg/g) was the maximum adsorption capacity.

The separation factor (R_L) for Langmuir model is used to evaluate the adsorption process, It showed that the isotherm was unfavorable (R_L>1), favorable (R_L<1), linear (R_L=1), or irreversible (R_L=0), which is defined as follow:

$$R_L = \frac{1}{1 + K_L C_m} \quad \text{(S5)}$$

Where C_m was the initial concentration.

2.2 Freundlich model:

$$Q_e = K_F {C_e}^{1/n} \quad \text{(S6)}$$

Where K_F and 1/n was the Freundlich constants.

2.3 Dubinin–Radushkevich model:

$$\ln Q_{eD} = \ln Q_D - B_D \varepsilon^2 \quad \text{(S7)}$$

$$\varepsilon = RT\ln(1 + \frac{1}{C_{eD}}) \quad \text{(S8)}$$

Where Q_{eD} was the maximum adsorption capacity (mmol/g), and BD was Dubinin–

Radushkevich isotherm constant (mol^2/kJ^2), R represented ideal gas constant (8.314 J/(mol·K)), T mean temperature (K), and C_{eD} represented equilibrium solution concentration (mmol/L).

The free adsorption energy (BD) was further used for the calculation of the mean adsorption energy (E, kJ/mol) to predict the occurrence of the ion-exchange mechanism by the following equation:

$$E=\frac{1}{\sqrt{2B_D}} \tag{S9}$$

The magnitude of 1 <E < 8 kJ/mol showed that the adsorption was mainly physical process, and the magnitude of 8 < E < 16 kJ/mol was mainly chemical adsorption.

Table S1. Content of major elements in cotton stalks.

Sample	C(%)	H(%)	O(%)	N(%)	S(%)	Fe(‰)	Si(‰)
Cotton stalks	44.85	5.73	44.74	0.95	0.37	0.44	1.56

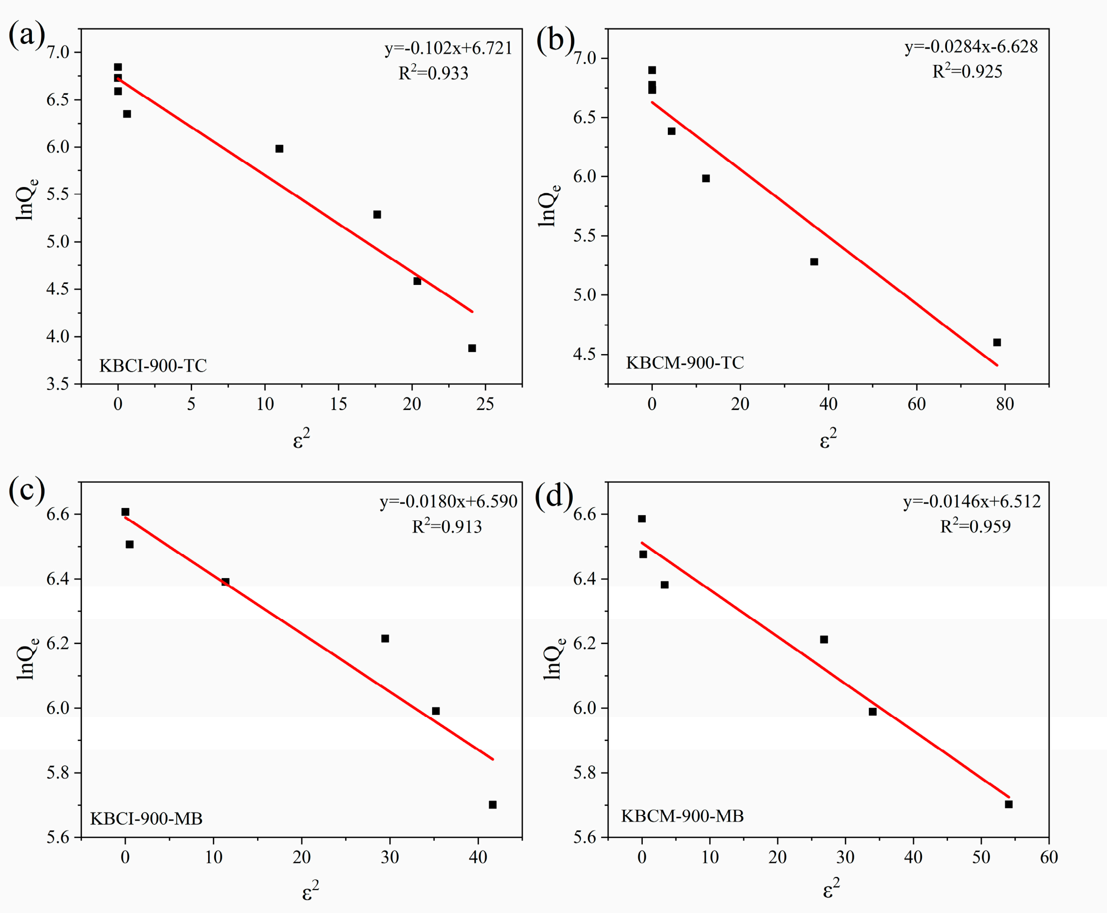

Figure S1. (a,b) D-R modeling plots of (a,b) KBCI-900 and (c,d) KBCM-900 for TC and MB

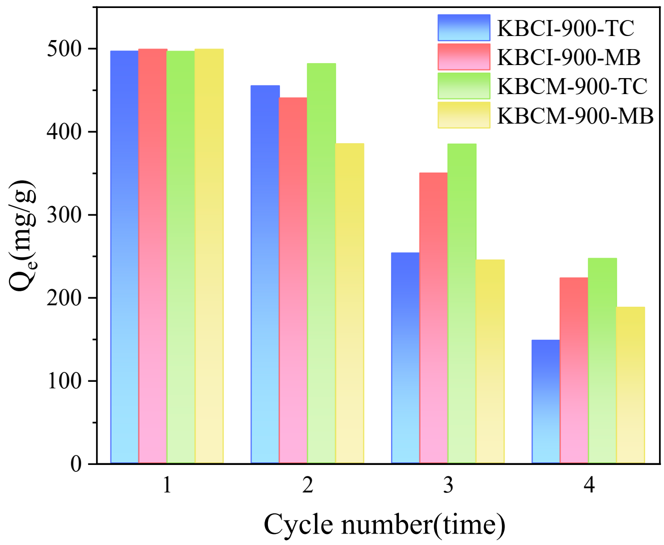

Figure S2. The 4 times of adsorption-desorption experiments by KBCI-900/KBCM-900.
